# Rhythmic auditory stimuli modulate movement recovery in response to perturbation during locomotion

**DOI:** 10.1242/jeb.237073

**Published:** 2021-03-01

**Authors:** Deepak K. Ravi, Marc Bartholet, Andreas Skiadopoulos, Jenny A. Kent, Jordan Wickstrom, William R. Taylor, Navrag B. Singh, Nick Stergiou

**Affiliations:** 1 Institute for Biomechanics, ETH Zürich, 8093 Zürich, Switzerland; 2 Department of Biomechanics and Center for Research in Human Movement Variability, University of Nebraska at Omaha, Omaha, NE 68182, USA; 3 Department of Environmental Agricultural and Occupational Health, University of Nebraska Medical Center, Omaha, NE 68198-4388, USA

**Keywords:** Recovery potential, Adaptive capacity, Physiological resilience, Aging, Non-linear dynamics, 1/*f* structure

## Abstract

The capacity to recover after a perturbation is a well-known intrinsic property of physiological systems, including the locomotor system, and can be termed ‘resilience’*.* Despite an abundance of metrics proposed to measure the complex dynamics of bipedal locomotion, analytical tools for quantifying resilience are lacking. Here, we introduce a novel method to directly quantify resilience to perturbations during locomotion. We examined the extent to which synchronizing stepping with two different temporal structured auditory stimuli (periodic and 1/*f* structure) during walking modulates resilience to a large unexpected perturbation. Recovery time after perturbation was calculated from the horizontal velocity of the body's center of mass. Our results indicate that synchronizing stepping with a 1/*f* stimulus elicited greater resilience to mechanical perturbations during walking compared with the periodic stimulus (3.3 s faster). Our proposed method may help to gain a comprehensive understanding of movement recovery behavior of humans and other animals in their ecological contexts.

## Abstract (precis)

**Summary:** A new method for the evaluation of intrinsic resilience during unsteady locomotion in humans and animals, analysing the relationship between the structure of movement variability and resilience.

## INTRODUCTION

Humans generally exhibit quick and accurate movement recovery to unexpected perturbations to facilitate stable walking (i.e. fall avoidance) while traversing real-world environments. The mechanics and control underlying recovery of movement and stable locomotion in humans are only now starting to be unraveled. Perturbation experiments on model locomotor systems (e.g. birds: Daley, 2018; Daley and Biewener, 2006; dogs: Wilshin et al., 2017; and human runners: Grimmer et al., 2008; Seethapathi and Srinivasan, 2019; Seyfarth et al., 2003) have further advanced our understanding, but have also revealed additional factors to consider. First, the assessment of movement recovery might depend on the organization level of the body (e.g. whole-body, joint or limb, and muscle level) at which recovery is estimated. For instance, corrective responses at the joint level can occur with minimal effect on the whole-body center of mass (COM) trajectory (Chang et al., 2009). Second, the form and function of an animal may determine the control requirements for maintaining stability (Daley, 2018; More and Donelan, 2018). For example, humans and some birds appear to use similar feedforward control (e.g. leg retraction) to counteract terrain perturbations during running (Muller et al., 2016; Seyfarth et al., 2003; Daley et al., 2007). However, reflex-mediated feedback control (of, for example, foot placement: Ignasiak et al., 2019; Joshi and Srinivasan, 2019) may be predominantly involved in locomotion at slow speeds (Marigold and Patla, 2005). Furthermore, it remains unclear how these mechanisms vary across different perturbation types and sizes.

Third (the point on which this paper builds), it has been extremely challenging to quantify the recovery of body dynamics during locomotion, as standard stability analyses and measures are based on no perturbations or small local perturbations, e.g. small differences in surface height or neuromuscular noise (Daley, 2016; Hamacher et al., 2011; Bruijn et al., 2013; van Emmerik et al., 2016). Small perturbations generally cause little or no noticeable deviation of body dynamics, while large perturbations (e.g. waist pulls or a large pothole in the terrain) instantaneously disrupt the inherent movement patterns of locomotion and invoke a change in walking behavior (trip or slip). The effects of small perturbations, if not attenuated, can also add up to become a large perturbation (Kurz and Stergiou, 2005; Su and Dingwell, 2007). Importantly, the adverse effects of a large perturbation generally persist over an extended period (Hof et al., 2010; Qiao and Jindrich, 2014; Jindrich and Full, 2002; Daley and Biewener, 2011), even though there may be no apparent residual feeling of instability. Answering the key question ‘How long does it take to recover movement after a perturbation?’ seems to be essential in order to provide an unambiguous and intuitive indicator of adaptive capacity of an individual. We propose that this quantity be termed ‘resilience’ (a term most prominently employed in literature in psychology, engineering and ecology: Gunderson, 2000; Scheffer et al., 2018; Hadley et al., 2017) to highlight its significance within locomotor systems. Our study also proposes a framework and a novel methodology to quantify resilience to perturbations by: (1) identifying variables that represent the movement of interest, (2) accurately portraying steady-state behavior associated with those variables, (3) inducing perturbations that markedly disrupt this steady-state behavior, and (4) determining the length of time it takes to return to steady-state behavior.

**Fig. 1.**
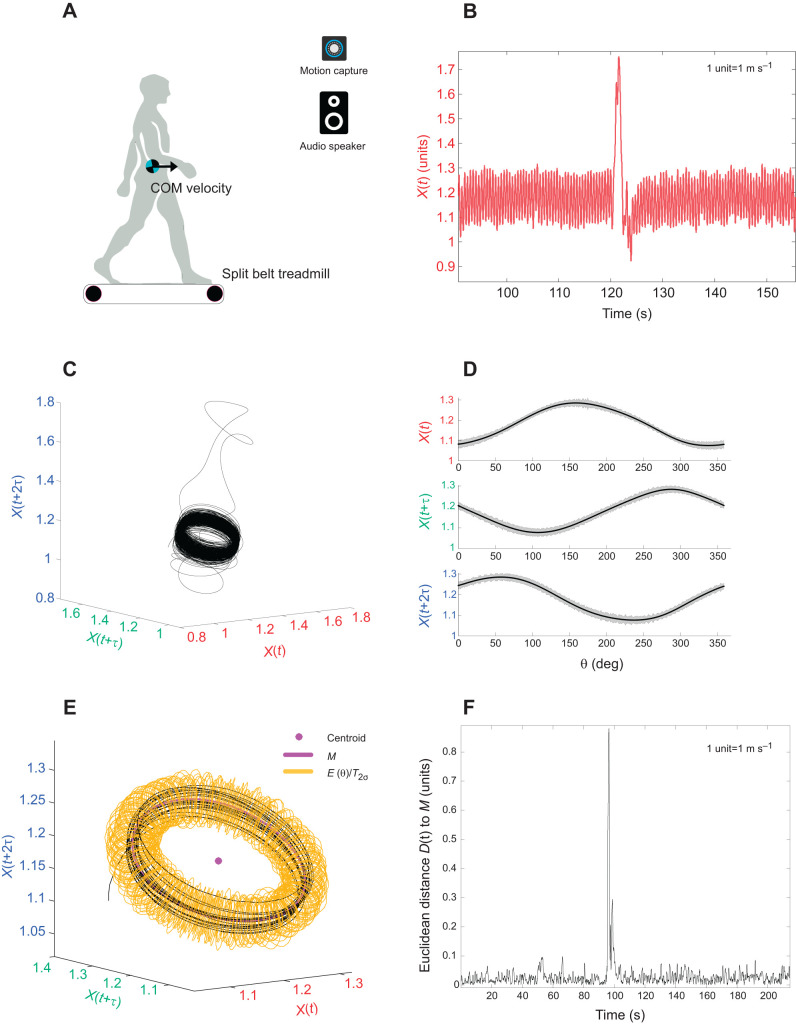
**Step-wise procedure for quantifying resilience.** An example is shown for a participant from the 1/*f* group. (A) Experimental setup. (B) Anterior–posterior center of mass (COM) velocity. *X*(*t*), COM velocity time series. (C) State-space reconstruction of time series [*X*(*t*), *X*(*t*+τ) and *X*(*t*+2τ), where τ is the time lag]. (D) Mean (solid line) and standard deviation (shaded area) of pre-perturbation reconstructed data in the three dimensions at every integer angle (θ) between 0 and 359 deg. (E) Construction of ellipses (*E*) (*n*=360) around the reference trajectory (*M*). The arrangement of ellipses resembles a torus. The torus *T*_2σ_ is shown here. (F) Euclidean distance between every attractor data point and reference trajectory.

Central to the quantification of resilience is the appropriate characterization of the steady-state behavior to which a human or animal may return after a perturbation (Full et al., 2002). To facilitate a stable steady locomotion, a strategy during walking and running could be to regulate the body's COM (Qiao and Jindrich, 2014; Hof, 2008; Blickhan and Full, 1993; Vlutters et al., 2016). State-space reconstruction of movement time series, e.g. COM, offers a representation of the underlying dynamics, as well as a geometric illustration of intrinsic steady-state behavior. During walking in humans, for example, the COM appears to describe a variable but also well-defined ring torus in three-dimensional state space (Fig. 1). Here, the apparent shape of the reconstructed time series provides geometric and statistical boundaries of steady-state behavior. We propose that an individual's resilience may be related to their ability to return to, and remain within, the boundaries of their steady-state behavior following a disturbance. Therefore, we quantify resilience as the time period from the instance of perturbation until steady-state movement patterns are once again achieved.

The steady-state behavior of locomotion displays structured patterns of stride-to-stride variability in humans (Hausdorff, 2007) and animals (Kyvelidou and Decker, 2016). For healthy systems, these patterns generally feature a 1/*f* frequency spectrum (i.e. power spectral density is inversely proportional to the frequency of the signal) (Goldberger et al., 2002; Stergiou and Decker, 2011). Early evidence suggests that stride-to-stride fluctuations exhibiting a 1/*f* structure of variability could facilitate stable and flexible locomotion (Stergiou and Decker, 2011; Ahn and Hogan, 2013). Conversely, deviations away from a 1/*f* structure may be indicative of either behavioral randomness (unstable and unpredictable) or periodic (non-flexible) behavior (Li et al., 2019). Both of these extremes may also characterize locomoting systems with low resilience that are less capable of withstanding perturbations, e.g. frailty in the elderly (Ravi et al., 2020). Furthermore, recent studies have demonstrated that the steady-state behavior of locomotion and its structured patterns of stride-to-stride variability can be manipulated by synchronizing walking with external auditory stimuli (Hunt et al., 2014; Vaz et al., 2020). This suggests that there may be a relationship between the structure of movement variability and resilience.

To test this, in our study we induced an unexpected perturbation in healthy, young adults as they synchronized their walking patterns to external auditory stimuli composed of either a periodic or 1/*f* structure. We anticipated that synchronizing to a periodic stimulus would artificially degrade the structure of variability in stride-to-stride walking patterns and induce non-flexible movement, resulting in a prolonged recovery time. In contrast, we expected that synchronizing to a 1/*f* stimulus would probably produce a more stable and flexible movement behavior as commonly observed in healthy systems (Hunt et al., 2014), resulting in a reduced recovery time. In this manner, we aimed to isolate the effect of the structure of movement variability on the ability to withstand perturbations by measuring the time taken to recover using the velocity of the COM time series.

## MATERIALS AND METHODS

Fifteen healthy young adults (*N*=6 females and *N*=9 males, age 19–30 years; height 1.76±0.18 m, mass 72.5±7.5 kg, means±s.d.) with no history of neurological, vestibular or movement disorders or other problems that could alter typical walking patterns participated in this study. The protocol was approved by the local institutional review board (protocol #189-16-EP). All subjects provided written informed consent prior to participating. In-depth study methodology can be found online at protocols.io (https://dx.doi.org/10.17504/protocols.io.bmu3k6yn) and a brief summary is provided below.

Subjects participated in two walking trials. For the first trial (‘baseline’), subjects walked on a split-belt treadmill (Bertec Corp.; Fig. 1A), secured with a harness (zero bodyweight support), at their preferred walking speed for 25 min.

After a 20 min seated rest break, subjects were randomly assigned to one of two rhythmic auditory stimulus conditions for their second trial (‘cued’): periodic (*N*=7) or 1/*f* (*N*=8). For the periodic condition, stimuli were set with an interbeat interval that matched the participants' mean interstride interval, obtained from the final 3 min of the baseline trial. 1/*f* time series were generated using custom-written Matlab code and similarly normalized to the mean and variance of the interstride interval obtained from the baseline walking trials. The stimulus patterns were translated into four-beat drum patterns within which every beat was sounded by a closed hi-hat and the first and third of every four beats were sounded by a bass drum and snare drum, respectively (see Audio 1 and 2 for examples). Subjects were asked to walk in synchrony with the strong beats of the drum pattern for 45 min. Twenty-five minutes into this trial, the treadmill belt of the non-dominant leg was arrested for 500 ms, delivering a brief unexpected perturbation that emulated a real-world trip. This occurred at the instant at which the ankle of the dominant limb in swing passed the ankle of the support limb in stance. After normal belt movement had resumed, the subjects were asked to ‘please keep walking’ and the trial continued for a further 20 min. The auditory stimuli were presented continuously, irrespective of the perturbation. Kinematic data from a full body marker set were recorded at 100 Hz using a 3D motion capture system (Vicon Motion Systems). The horizontal velocity of the COM (anterior–posterior) was calculated from 2 min before to 2 min after the perturbation. The velocity of the base of support (i.e. equal to the velocity of the treadmill) was added to COM velocity (Fig. 1B).

Our method to quantify resilience to perturbations during locomotion involved the steps detailed below in ‘Determination of intrinsic steady state’ and ‘Quantification of post-perturbation recovery time'.

### Determination of intrinsic steady state

(1) The COM velocity time series *X*(*t*) was reconstructed using the time delay embedding method (readers are referred to Wurdeman, 2016, for further reading) involving an embedding dimension (*d*) and time lag (τ) (Fig. 1C). The state space vector [*X*(*t*), *X*(*t*+τ), *X*(*t*+2τ)] was then divided into pre- and post-perturbation phases. (2) The centroid of the pre-perturbation vector was found by taking the mean of the attractor data points. A reference trajectory (*M*) was fitted using an eight-term Fourier model (Fig. 1D; Table S1 for details on fitting statistics). (3) For every attractor data point in the state space vector, the corresponding angle around the centroid and its Euclidean distance *D*(*t*) to *M* was determined. Around the reference trajectory, an ellipse at each integer angle (θ) between 0 and 359 deg was constructed (Fig. 1E) as follows. (i) The length of the semi-major axis of the ellipse was equal to the largest standard deviation of the enclosed data points from the three dimensions. The second largest standard deviation gave the length of the semi-minor axis. Each ellipse was defined using 50 points (see Fig. S1A). (ii) The center of the ellipse coincided with *M*, while the semi-major axis was oriented toward the data point furthest away from *M*. The placement of the ellipses around the reference trajectory thus resembled a torus *T*_1σ_. Please refer to Fig. S1B for a step by step illustration of the ellipse construction. (iii) This computation was repeated to construct *T*_2σ_ and *T*_3σ_ using two and three times the standard deviation of the data, respectively.

### Quantification of post-perturbation recovery time

**Fig. 2.**
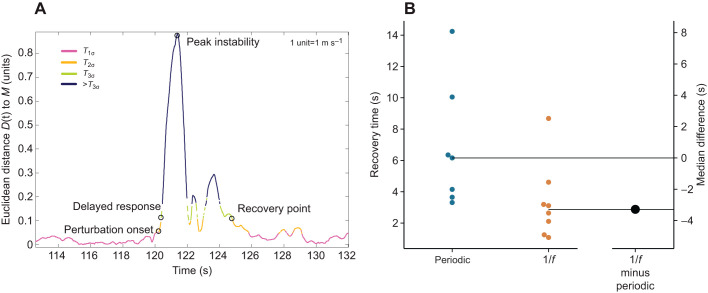
**Resilience characteristics.** (A) Quantification of recovery time to steady-state patterns. (B) Gardner–Altman plot showing the recovery time of all participants (*N*=15) and the median difference between the periodic (*N*=7) and 1/*f* group (*N*=8). Significant differences (*P*<0.05) between the conditions as determined by the Wilcoxon rank-sum test.

(1) The position of each data point was labeled according to the smallest constructed torus, *T*_1σ_, *T*_2σ_ or *T*_3σ_, that enclosed the data (Figs 1F and 2A). In the current study, torus *T*_2σ_ was assumed to indicate the boundary for steady-state behavior and used for further analysis. (2) The point of recovery was defined as the time point after which the trajectory no longer left the torus for five consecutive walking cycles, permitting four outliers lasting no more than 10 ms each (Movies 1 and 2). The time to recover to steady-state movement (‘recovery time’) was calculated as the period between the instant of perturbation and the point of recovery.

### Analyses

Medians are provided for each stimulus condition and visualized using a Gardner–Altman estimation plot. The Wilcoxon rank sum test was used to compare the stimulus groups based on the calculated recovery time. Recovery time could potentially be influenced by walking speed. We thus also compared walking speed between the groups. Statistical significance was determined at *P*<0.05.

All analyses were conducted in Matlab (v2019a, The Mathworks Inc.).

## RESULTS AND DISCUSSION

Our work introduces a new method to quantify resilience to large perturbations during human and animal locomotion. Additionally, we investigated the relationship between the structure of movement variability and resilience by having our participants synchronize their walking patterns to one of two auditory stimuli – (1) a non-variable periodic stimulus or (2) a variable stimulus with 1/*f* structure – and observed their time to recover pre-perturbation steady-state movement patterns. The 1/*f* group exhibited a faster recovery after perturbation compared with the periodic group, with a difference in median recovery time of 3.3 s (periodic: 6.16 s versus 1/*f*: 2.88 s, medians, *W*=76, *P*=0.021; Fig. 2B). There was no difference between the groups for walking speed (periodic: 1.15±0.06 m s^−1^ versus 1/*f*: 1.15±0.14 m s^−1^, means±s.d., *W*=54.5, *P*=0.889). Thus, the quantification of an individual's recovery time to a steady-state movement pattern after perturbation provided a strong conceptual framework for assessing resilience in locomotion.

Regulation of movement to respond rapidly and appropriately to unexpected perturbations is achieved by the interaction and integration of sensory information, musculoskeletal properties and motor networks (Nishikawa et al., 2007). Physiological outputs such as stride-to-stride walking variability exhibit 1/*f* structure and it is believed to reflect the complexity and integrity of movement regulation (Lipsitz, 2002). Complementing existing literature that elucidates the significance of 1/*f* structure of movement variability to health and function (Hausdorff, 2007; Goldberger et al., 2002), our results provide the first empirical evidence that it may also enable greater resilience to perturbations. It has been suggested that 1/*f* stimulus (a) primes the walking system to variations and may build up a repertoire of locomotor patterns that would allow faster recovery from perturbations (West, 1990; Cavanaugh et al., 2017; Diniz et al., 2011) and (b) promotes interactivity among subsystems and high synchrony with other biorhythms to build adaptive capacity of the animal (Harrison et al., 2018).

To date, the hypotheses of the effect of alterations in temporal structure of movement variability on the ability of an individual to withstand perturbations that could lead to a fall have not been investigated. This may be due, in part, to the difficulty of employing perturbation analysis in a clinical setting with vulnerable populations in a safe manner. Our study showcased an experimental paradigm to emulate vulnerable populations without recruiting and conducting tests on them first. As such, the study revealed that synchronizing to a periodic stimulus artificially degraded the structure of walking patterns and resulted in a longer recovery time compared with the 1/*f* group. It would be interesting in the future to confirm our results in individuals suffering from movement disorders who walk in a naturally periodic-like manner and have increased rigidity. In such populations, 1/*f* stimulus intervention may be pursued in a within-subject design to evaluate its effects on improving resilience over and above the baseline status (no stimulus). Furthermore, a natural question is whether an individual is really less stable during the long recovery period. Thus, it is important for future research to extend our protocol to determine failure (causing a fall) and learning effects using multiple perturbations. To consistently assess these characteristics across individuals, perturbations to a subject should be applied at the same instant of the recovery period (to assess failure) or after full recovery (to assess learning; Bruijn et al., 2013; Pater et al., 2015). We see an advantage to using our method, as it can provide these characteristics across individuals and help standardize protocols. Our study results are also important because periodic cueing is often used in movement rehabilitation (Damm et al., 2020).

Our method can uniquely uncover behaviorally relevant information of recovery time to steady-state patterns after large perturbations that are difficult to compute using existing approaches (Bruijn et al., 2013; Hamacher et al., 2011). Moreover, only a few existing measures apply outdoors and this probably makes the challenges for assessment in natural contexts especially acute for studying animals. Measures based on non-linear analysis such as gait sensitivity norm (Hobbelen and Wisse, 2007) and Floquet multipliers (Hurmuzlu and Basdogan, 1994) have been used to characterize response to perturbations during walking (Kang and Dingwell, 2008). However, such analyses quantify only discrete responses of gait parameters (observing only a particular event that occurs during a gait cycle, e.g. heel strike) to a perturbation and assume complete periodicity of the gait cycle (Dingwell et al., 2001; Bruijn et al., 2013). Other existing measures such as extrapolated COM (Hof, 2008) and Lyapunov stability analysis (Dingwell and Cusumano, 2000) have been used but once again not in a manner to explicitly compute recovery time after a large perturbation (Bierbaum et al., 2011; Vlutters et al., 2016; Stergiou et al., 2004). More general techniques based on stabilization of discrete gait parameters have been used (Rosenblum et al., 2020; McCrum et al., 2018), but ambiguities remain with respect to the appropriate characterization of steady-state behavior, limiting their usefulness. State-space reconstruction of time series marks the first and basic step in many of the above-mentioned non-linear tools applied to walking (Bruijn et al., 2013). As our method uses state-space reconstruction, we believe that in a foundational context, our method is essentially tied to other approaches inspired from a dynamical systems perspective with respect to perturbed walking and extends to large perturbations.

We only analyzed the time series of the COM velocity; however, our method can be applied to other sinusoidal systems (e.g. leg angular trajectories). This includes other cyclic movements (e.g. running, hopping), conditions (e.g. uneven terrain, obstacle negotiation) and biped locomotor systems (e.g. guineafowl, ostrich). Based on our results, we predict that in all these model systems, when they exhibit 1/*f* type movement patterns, they will be better at maintaining stability and recover faster after perturbations. However, a limitation to this torus-based approach is that there are other biomechanical variables whose state-space trajectories do not resemble a torus but might be indicative of a much less stable state after perturbation. At the conceptual level, our method assumes that an individual returns to the steady-state pattern immediately after a perturbation. However, in reality, it might be that the subject may take longer to return or may not return at all to the original steady state and might continue to move with a small persisting difference from the baseline level and with a new steady state (Daley, 2016; Hadley et al., 2017). At the computational level, the number of terms or harmonics used in the Fourier model to fit the reference trajectory, the point of recovery and other choices, if necessary, need to be tailored to individual participants. We invite future work to assess the sensitivity of our method to such differences. While resilience at the level of body kinematics is useful, these measures do not automatically reveal the neuromuscular mechanisms underlying the perturbation response. In future work, it will be interesting to investigate recovery time together with response latency (Zhang et al., 2020), sensorimotor delays (Daley, 2018) and/or regulation of muscle dynamics (Hof and Duysens, 2013). This exploration may help make sense of the sensorimotor systems or even task-level strategies (e.g. to avoid injury or use less energy) that regulate recovery time to steady state.

Overall, our approach offers the potential to be used in tandem with wearable devices, such as subject-borne accelerometers for on-the-fly, continuous evaluation of resilience during unsteady locomotion. For example, brief periods of observation of body COM during unperturbed locomotion would be first used to define the torus (similar to Fig. 1E). In the case of a perturbation (such as a human who has lost their balance on a patch of ice or an animal faced with an unexpected drop in terrain height), our method can determine the time to recover to the torus associated with their unperturbed state. Over time, a discernible trend towards slower recovery from a perturbation might go hand-in-hand with the onset of symptoms of a movement disorder or frailty (Ferrucci et al., 2018). As such, our approach could address long-standing and important challenges in human rehabilitation (e.g. fall risk identification), animal studies (e.g. in their ecological contexts) and bipedal robotics.
